# NR1D1 suppressed the growth of ovarian cancer by abrogating the JAK/STAT3 signaling pathway

**DOI:** 10.1186/s12885-021-08597-8

**Published:** 2021-07-30

**Authors:** Huailin Wang, Yan Fu

**Affiliations:** Department of Gynecology, the First Hospital of Jilin University, 71 Xinmin Avenue, Changchun, 130021 China

**Keywords:** Ovarian cancer, NR1D1, SOCS3, JAK/STAT3, Proliferation, Apoptosis

## Abstract

**Background:**

Nuclear receptor subfamily 1 group D member 1 (NR1D1), a nuclear receptor associated with a variety of physiological processes, has a low level in ovarian cancer tissues compared with adjacent normal tissues. However, its role in ovarian cancer remains unclear.

**Methods:**

The level of NR1D1 in ovarian cancer cells was determined by quantitative real-time PCR. Its role in ovarian cancer was explored through gain-of-function and lose-of-function. Cell growth was evaluated by CCK8 assay, immunofluorescence and flow cytometry. Western blot was conducted to assess the activation of JAK/STAT3 signaling pathway. A xenograft model of ovarian cancer was established to explore the role of NR1D1 in vivo.

**Results:**

Up-regulation of NR1D1 repressed the ovarian cancer cell proliferation and induced cell cycle arrest and apoptosis, while silencing NR1D1 promoted their proliferation and G1/S transition. In addition, the JAK/STAT3 signaling pathway, an intracellular signal transduction closely associated with cancer progression, was inhibited by NR1D1. Consistently, xenografts with NR1D1 over-expression grew more slowly in vivo than the controls. Furthermore, NR1D1 up-regulated the expression of suppressor of cytokine signaling 3 (SOCS3), an inhibitor of the JAK/STAT3 signaling pathway. Whereas, SOCS3 silencing abolished the function of NR1D1 over-expression on ovarian cancer growth and JAK/STAT3 signaling pathway.

**Conclusions:**

NR1D1 up-regulated the expression of SOCS3, resulting in suppression of the JAK/STAT3 signaling pathway, thus retarding the growth of ovarian cancer cells. This study highlights a profound role of NR1D1 in the treatment of ovarian cancer.

**Supplementary Information:**

The online version contains supplementary material available at 10.1186/s12885-021-08597-8.

## Background

Ovarian cancer is one of the most common gynecologic malignant cancers worldwide. There was an estimate of 22,240 new cases of ovarian cancer and 14,070 deaths in 2018 in the United States [1]. Cytoreductive surgery accompanied with adjuvant chemotherapy is the first-line treatment for ovarian cancer [2]. It leaves an enormous threat to the health care system due to its high incidence. Due to the lack of reliable early symptoms, the early diagnosis of ovarian cancer is difficult. Its 5-year-survival at distant-stage is nearly 30% [3]. Thus, it is necessary to have a better understanding of the molecular alterations in ovarian cancer to identify a novel target for early diagnosis and treatment.

Nuclear receptor subfamily 1 group D member 1 (NR1D1), also known as REV-ERBα, is a nuclear receptor involved in the regulation of several physiological processes [4, 5]. NR1D1 is also reported to be involved in cancers. Pharmacological activation of NR1D1 is lethal to many cancers [6, 7]. It can enhance DNA damage and suppress DNA repair [8, 9]. Additionally, NR1D1 is associated with chemosensitivity of breast cancer [8]. Its high expression has a favorable influence on the survival of patients with breast cancer [10]. According to data from gene expression profiling interactive analysis (GEPIA) website based on the Cancer Genome Atlas (TCGA) data, NR1D1 is lowly expressed in ovarian cancer tissues. However, its role in ovarian cancer remains unclear.

Janus kinase (JAK)/ signal transducer and activator of transcription (STAT) 3 signaling pathway, a famous intracellular signal transduction system, is implicated in various bioprocesses, including proliferation, cycle progress and survival [11]. In response to binding with cytokines or growth factors, these receptors coupled with JAKs undergo a conformational change, leading to the activation of JAKs [12]. Then the JAKs are cross-phosphorylated and phosphorylate the cytoplasmic domains of these receptors. Phosphorylated cytoplasmic domains serve as the STAT docking sites, resulting in STAT recruitment and phosphorylation. The phosphorylation of STATs leads to their conformational change and translocation to the nucleus where they perform their function as transcriptional factors regulating the expression of target genes [13]. Abnormal activation of the JAK/STAT3 signaling pathway, which contributes to the pathogenesis and progression of cancers [14–16], was also noted in ovarian cancer [17, 18]. Suppressing the JAK/STAT3 signal markedly reduces the tumor progression and metastasis of ovarian cancer [19]. Suppressor of cytokine signaling (SOCS) 3, an inhibitor of the JAK/STAT3 signaling pathway, is correlated to the pathogenesis and progression of multiple cancers and regarded as a crucial tumor suppressor [20–22]. SOCS3 obstructs STAT phosphorylation via repressing the activation of JAKs [23, 24]. Interestingly, the level of SOCS3 was positively correlated to NR1D1 in ovarian cancer according to data from GEPIA. However, whether SOCS3/JAK/STAT3 is implicated in the role of NR1D1 in ovarian cancer remains not yet clear.

Herein, we explored the function of NR1D1 on the growth of ovarian cancer cells and the activation of SOCS3/JAK/STAT3 signaling pathway. Our results highlight a profound role for NR1D1 in ovarian cancer.

## Methods

### Gene expression analysis

Online website GEPIA (http://gepia.cancer-pku.cn/) was used to analyze the NR1D1 expression in ovarian cancer tissues (*n* = 426) and normal ovarian tissues (*n* = 88) as well as its correlation to SOCS3 based on TCGA data. The Pearson method was employed in the determination of correlation coefficient. Kaplan-Meier plotter databases (https://kmplot.com/) was employed to generate the overall survival curves (*n* = 1145).

### Cell culture

Human ovarian cancer cell lines COC1 (Cat No. CL-0064; Procell, Shanghai, China), A2780 (Cat No. iCell-h004; iCell Bioscience, Shanghai, China) and human normal ovarian epithelial cells (NOEC; Cat No. iCell-h112; iCell Bioscience) were grown in RPMI-1640 (Gibco, Life Technologies, Saint-Aubin, France) with 10% fetal bovine serum (FBS; Hyclone, Logan, UT, USA). SK-OV-3 cells (Cat No. CL-0215; Procell) were grown in McCoy’s 5A (Procell) with 10% FBS. RPMI-1640 with 20% FBS was used in the culture of OVCAR-3 cells (Cat No. CL-0178; Procell) and DMEM (Gibco) with 10% FBS was employed in the culture of 293 T cells (Cat No. ZQ0033; Zhongqiaoxinzhou Biotechnology, Shanghai, China). All these cells were cultured in a humid atmosphere with 5% CO_2_ at 37 °C. All cells were mycoplasma–free and authenticated by short tandem repeat.

### Transfection

Cells seeded in 6-well plates (4 × 10^5^ cells/well) were transfected with NR1D1 over-expression plasmid (GeneScript, Nanjing, China), NR1D1 shRNAs or SOCS3 siRNAs using Lipofectamine 3000 Reagent (Invitrogen, ThermoFisher, Waltham, Massachusetts, USA) according to the protocol. G418 (300–400 μg/ml) was added into cells for the selection of stably transfected cells. The sequences (5′- > 3′) for NR1D1 shRNAs or SOCS3 siRNAs were as follows:

- NR1D1 shRNA-1: GCCCTGAATCCCTCTATAGTTTCAAGAGAACTATAGAGGGATTCAGGGTTTTT;
- NR1D1 shRNA-2: GGCAACATCACCAAGCTGAATTCAAGAGATTCAGCTTGGTGATGTTGCTTTTT;
- NR1D1 shRNA-3: GGTCATAACGAGGCCCTAAATTCAAGAGAUTTAGGGCCTCGTTATGACTTTTT;
- SOCS3-siRNA-1: sense: CCCAGAAGAGCCUAUUACATT; anti-sense: UGUAAUAGGCUCUUCUGGGTT;
- SOCS3-siRNA-2: sense: UGGUCACCCACAGCAAGUUTT; anti-sense: AACUUGCUGUGGGUGACCATT’;
- SOCS3-siRNA-3: sense: UGGCCACUCUUCAGCAUCUTT; anti-sense: AGAUGCUGAAGAGUGGCCATT;
- Si-NC; sense: UUCUCCGAACGUGUCACGUTT; anti-sense: ACGUGACACGUUCGGAGAATT.

### Quantitative real-time PCR (qRT-PCR)

A high-purity RNA extraction kit (BioTeke, Beijing, China) was used to extract the total RNA. The first strand of cDNA was synthesized using M-MLV reverse transcriptase (TaKaRa Bio, Shiga, Japan). qRT-PCR was performed to determine the mRNA levels of NR1D1 and SOCS3. The primers (5′- > 3′) used were listed below:

- NR1D1 forward: CCCTGGGAGTCTACAAGTGG;
- NR1D1 reverse: GCGATTGATGCGGACGAT;
- SOCS3 forward: TCGCCACCTACTGAACCCT;
- SOCS3 reverse: GGTCCAGGAACTCCCGAAT
- β-Actin forward: GGCACCCAGCACAATGAA;
- β-Actin reverse: TAGAAGCATTTGCGGTGG.
- β-Actin served as the internal reference. 2^-ΔΔCt^ method was employed to calculate the relative level of target mRNA .

### Western blot

RIPA lysis buffer (with 1% phenylmethanesulfonyl fluoride) (Beyotime, Shanghai, China) was used to lyse the cells. After determination of protein concentration with a BCA protein concentration determination kit (Beyotime), the protein samples were subjected to sodium dodecyl sulfate-polyacrylamide gel electrophoresis followed by transferation onto polyvinylidene fluoride membranes (ThermoFisher). Following blocking with 5% bovine serum albumin, the membranes were incubated with antibodies against NR1D1 (1:1000; Abclonal, Wuhan, China), cyclinD (1:1000; ABclonal), cyclinE (1:1000; Proteintech, Wuhan, China), SOCS3 (1:1000; ABclonal), JAK-1 (1:1000; Affinity, Changzhou, China), p-JAK1 (Tyr 1034/Tyr 1035; 1:1000; Affinity), JAK2 (1:500; Affinity), p-JAK2 (Tyr 1007/Tyr 1008, 1:1000; Affinity), STAT3 (1:500; Affinity), p-STAT3 (Tyr 705, 1:500; Affinity), β-actin (1:2000; Proteintech) at 4 °C overnight. Thereafter, the membranes were incubated with horseradish peroxidase-labeled secondary antibodies (1:10000; Proteintech) at 37 °C for 40 min. Blots were visualized with an enhanced chemiluminescence substrate kit (7 Sea biotech, Shanghai, China).

### Cell viability assay

The cell viability was determined by cell counting kit-8 (CCK-8) (Sigma, St. Louis, MO, USA). Cells seeded in 96-well plates (4 × 10^3^ cells/ well) in quintuplicate were cultured in a cell incubator. At 0 h, 6 h, 24 h, 48 h and 72 h, CCK-8 (10 μl) was added into cells and incubated for 1 h. A microplate reader (BIOTEK, Winooski, VT, USA) was used to determine the absorbance at 450 nm.

### Immunofluorescence

Paraffin-embedded tumors were cut into 5 μm-sections. Then the sections were deparaffinated, rehydrated and antigen-retrieved. Cells were seeded onto coverslips. Forty-eight hours later, the cells were fixed in 4% paraformaldehyde, followed by permeabilizing in 0.1% TritonX-100. Incubation with goat serum (Solarbio, Beijing, China) was performed to block non-specific sites. Then the sections were incubated with antibodies against PCNA (1:200; Proteintech) or Ki-67(1:100; ABclonal) at 4 °C overnight, followed by incubating with Cy3-conjudged secondary antibody (1:200; Beyotime). Nucleus was stained with DAPI. Images were captured under a fluorescence microscope (OLYMPUS, Tokyo, Japan).

### Flow cytometry

For cell cycle determination, the cells were fixed in 70% ice-cold ethanol. After washing in PBS, the cells were stained with propidium iodide and RNaseA in the cell cycle determination kit (Beyotime) at 37 °C for 30 min. Then the cells were analyzed with a flow cytometry (NovoCyte, ACEA Biosciences, San Diego, CA, USA).

For the determination of cell apoptosis, the cells were stained with a cell apoptosis determination kit (KeyGen, Nanjing, China) at room temperature for 15 min. Then the cells analyzed with a flow cytometry.

### Activities of caspase-3 and caspase-9

Cells were harvested and lysed. The levels of activated of caspase-3 and caspase-9 in cells were determined with a caspase-3 activity determination kit (Beyotime) or caspase-9 activity determination kit (Solarbio) according to the instructions.

### Animal experiment protocols

BALB/c nude mice (4-week-old; Huafukang Bioscience, Beijing, China) were fed in a standard condition (12 h-light/dark cycles, 21–23 °C, 45–55% humidity). 1 × 10^6^ OVCAR-3 cells with stably transfection of NR1D1 over-expression plasmid were subcutaneously injected into the left flank (*n* = 6). Twenty-one days later, the mice were sacrificed and then tumors were harvested for subsequent hematoxylin-eosin (HE) staining, terminal deoxynucleotidyl transferase-mediated dUTP nick end labeling (TUNEL) assay and western blot. All the animal experimental protocols were in accordance with the Guide for Care and Use of Laboratory Animals and approved by the Ethics Committee of the First Hospital of Jilin University (20200698).

### HE staining

Paraffin-embedded tumors were cut into 5 μm-sections. After deparaffinization and rehydration, the sections were subjected to routine HE staining.

### TUNEL assay

TUNEL assay was performed using an in situ cell death detection kit (Roche, Penzberg, Germany). After deparaffinization and rehydration, the sections were permeabilized in 0.1% TritonX-100, blocked with 3% hydrogen peroxide and then stained with TUNEL reaction solution in the dark for 1 h. Thereafter, the sections were incubated with Converter-POD solution at 37 °C for 30 min and visualized with a DAB substrate kit (Solarbio) and counterstained with hematoxylin. Images were captured under a microscope (OLUMPUS).

### Statistical analysis

Data were presented as mean + standard deviation (SD). Student’s t test or one-way analysis of variance followed by Tukey’s multiple comparison as the post-hoc were used in data analysis. *P* < 0.05 was considered as significant difference.

## Results

### NR1D1 inhibited the proliferation of ovarian cancer cells

**Fig. 1:**
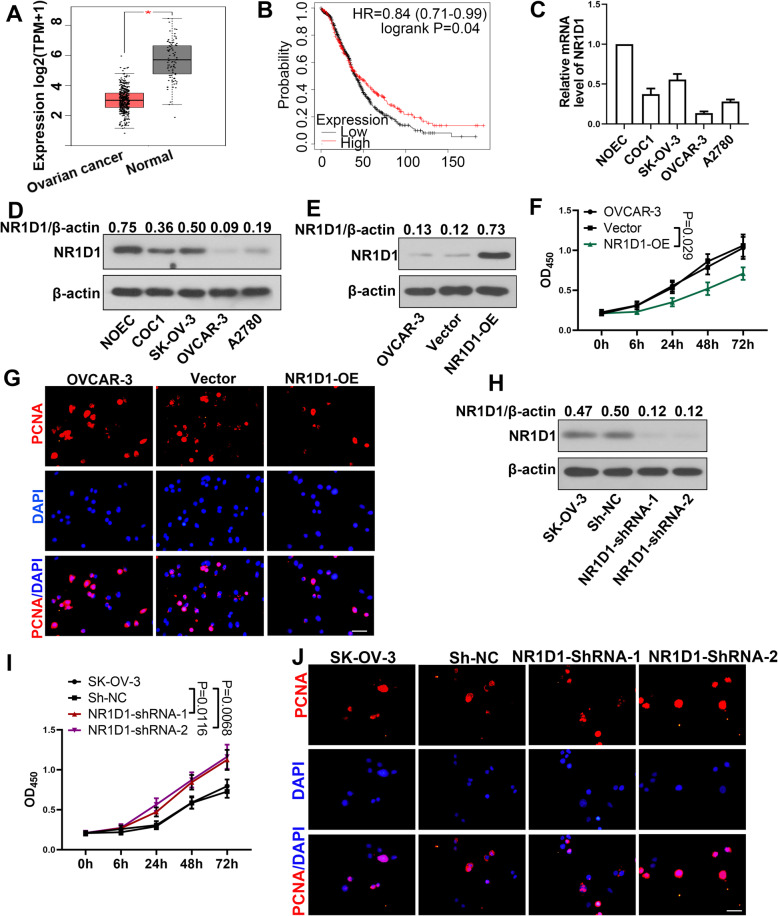
NR1D1 inhibited the proliferation of ovarian cancer cells. **A** Data from TCGA showed that NR1D1 has a low expression level in ovarian cancer tissues (ovarian cancer *n* = 426 and normal ovarian tissues *n* = 88). **B** Low NR1D1 level was correlated to the poor survival of ovarian cancer patients at advanced stage (*n* = 1145). **C-D** The level of NR1D1 in ovarian cancer cell lines COC1, SK-OV-3, OVCAR-3, A2780 and normal ovarian epithelial cell line (NOEC) was determined by quantitative real-time PCR and western blot. **E** Transfection efficiency of NR1D1 over-expression plasmid was determined by western blot. **F** After transfection with NR1D1 over-expression plasmid, the proliferation of ovarian cancer cells was determined with CCK-8. **G** The level of PCNA in ovarian cancer cells was determined by immunofluorescence after transfection with NR1D1 over-expression plasmid. Scale bar = 50 μm. **H** The transfection efficiencies of NR1D1 shRNAs in ovarian cancer cells were determined by western blot. **I** The proliferation of NR1D1 silenced cells was determined with CCK-8. (J) Immunofluorescence was performed to determine the level of PCNA in NR1D1 silenced cells. Scale bar = 50 μm. The results are presented as mean + SD

To explored the function of NR1D1 in ovarian cancer, we employed GEPIA website to analyze the NR1D1 expression in ovarian cancer tissues (*n* = 426) and normal tissues (*n* = 88). Based on data from TCGA, the GEPIA website showed that NR1D1 expression in ovarian cancer is lower than the normal tissues (Fig. 1A). Additionally, the Kaplan-Meier plotter databases showed that the low NR1D1 expression was associated with poor survival at advanced stages (*n* = 1145) (Fig. 1B). Herein, we investigated the function of NR1D1 in ovarian cancer. First, the NR1D1 level in ovarian cancer cell lines COC1, SK-OV3, OVCAR3, A2780 and a normal ovarian epithelial cell NOEC was determined by qRT-PCR and western blot. Ovarian cancer cell lines showed a lower NR1D1 level than NOEC (Fig. 1C-D). Thereafter, a NR1D1 over-expression plasmid was transfected into OVCAR3 cells, which showed the lowest NR1D1 level, to explore the role of NR1D1. In cells transfected with NR1D1 over-expression plasmid, the level of NR1D1 was increased (Fig. 1E). In addition, the proliferation of NR1D1 over-expressed cells was slower than cells transfected with vector (Fig. 1F). The level of PCNA, a biomarker to evaluate the cell proliferation, was also declined in NR1D1 over-expressed cells (Fig. 1G). In addition, shRNAs for NR1D1 were transfected into SK-OV-3 cells, which showed the highest NR1D1 level in ovarian cancer cell lines. NR1D1 shRNAs decreased the level of NR1D1 in ovarian cancer cells (Fig. 1H). Meanwhile, the proliferation of NR1D1 silenced cells was accelerated (Fig. 1I), with an increased PCNA level (Fig. 1J). These results suggested that NR1D1 inhibited the proliferation of ovarian cancer cells.

### NR1D1 induced cell cycle arrest and apoptosis in ovarian cancer cells

**Fig. 2:**
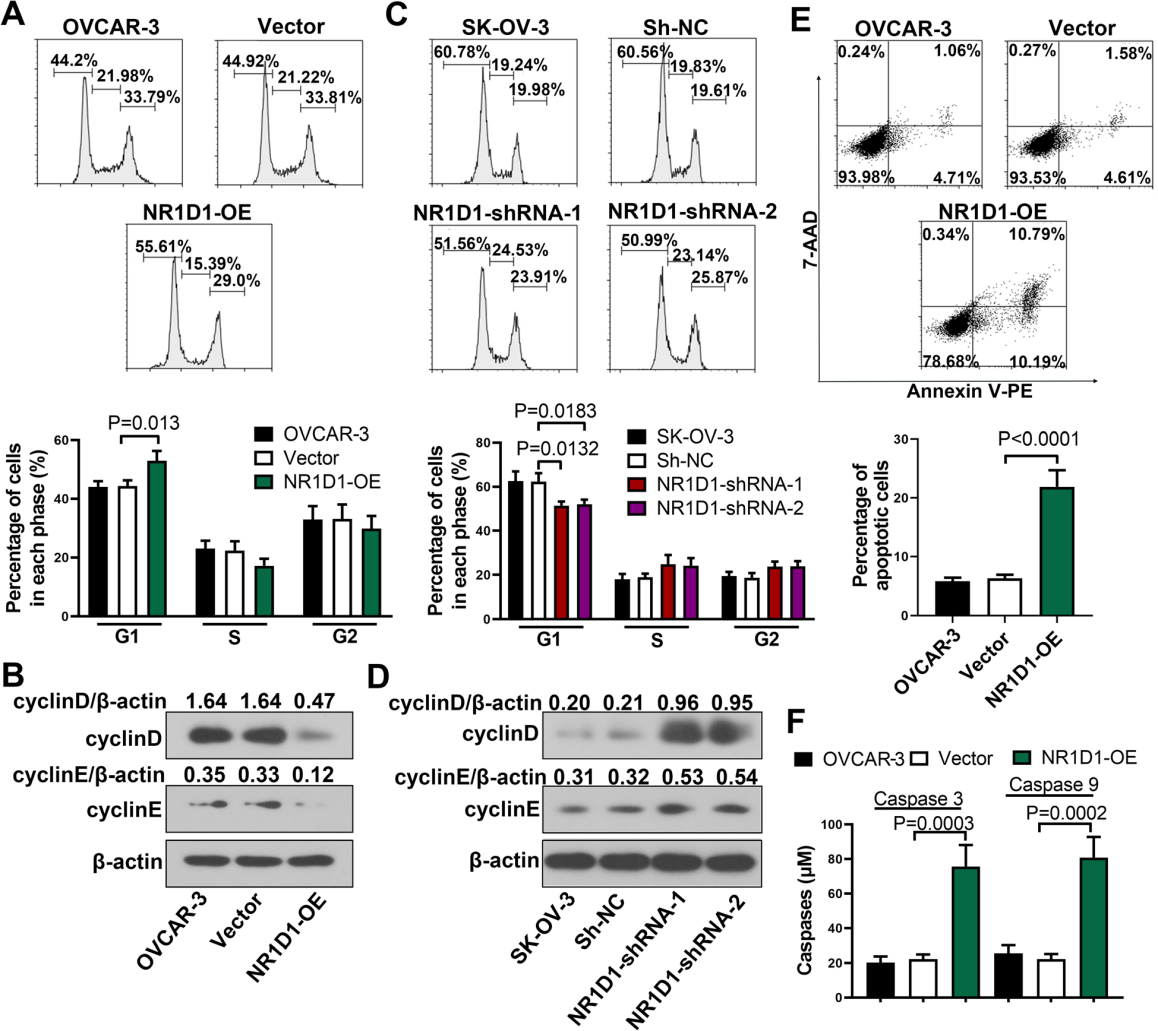
NR1D1 induced cell cycle arrest and apoptosis in ovarian cancer cells. **A** Cell cycle of NR1D1 over-expressed cells was determined by flow cytometry. **B** The levels of cyclinD and cyclinE were determined by western blot. **C** Flow cytometry was performed to determine the cell cycle of NR1D1 silenced cells. **D** Western blot was performed to determine the levels of cyclinD and cyclinE. **E** Apoptosis of NR1D1 over-expressed cells was determined by flow cytometry. **F** After transfection with NR1D1 over-expression plasmid, the levels of activated caspase-3 and caspase-9 were determined. The results are presented as mean + SD

Cell cycle and apoptosis are important factors impacting the cell growth. The role of NR1D1 in the cell cycle was further explored by flow cytometry. In NR1D1 over-expressed cells, the percentage of cells in G1 phase was increased (Fig. 2A). The levels of cyclinD and cyclinE, which were important factors controlling G1/S transition, were also declined in NR1D1 over-expressed cells (Fig. 2B). In addition, in NR1D1 silenced cells, the percentage of cells in G1 phase was decreased (Fig. 2C), and the levels of cyclinD and cyclinE were increased (Fig. 2D). These results indicated that NR1D1 retarded cell cycle of ovarian cancer cells.

Furthermore, the function of NR1D1 in cell apoptosis was also explored. In NR1D1 over-expressed cells, the percentage of apoptotic cells was increased (Fig. 2E). The activities of caspase-3 and caspase-9, which were important biomarkers for apoptosis, were also increased in NR1D1 over-expressed cells (Fig. 2F). These results suggested that NR1D1 induced apoptosis of ovarian cancer cells.

### NR1D1 inhibited the activation of JAK/STAT3 signaling pathway

**Fig. 3:**
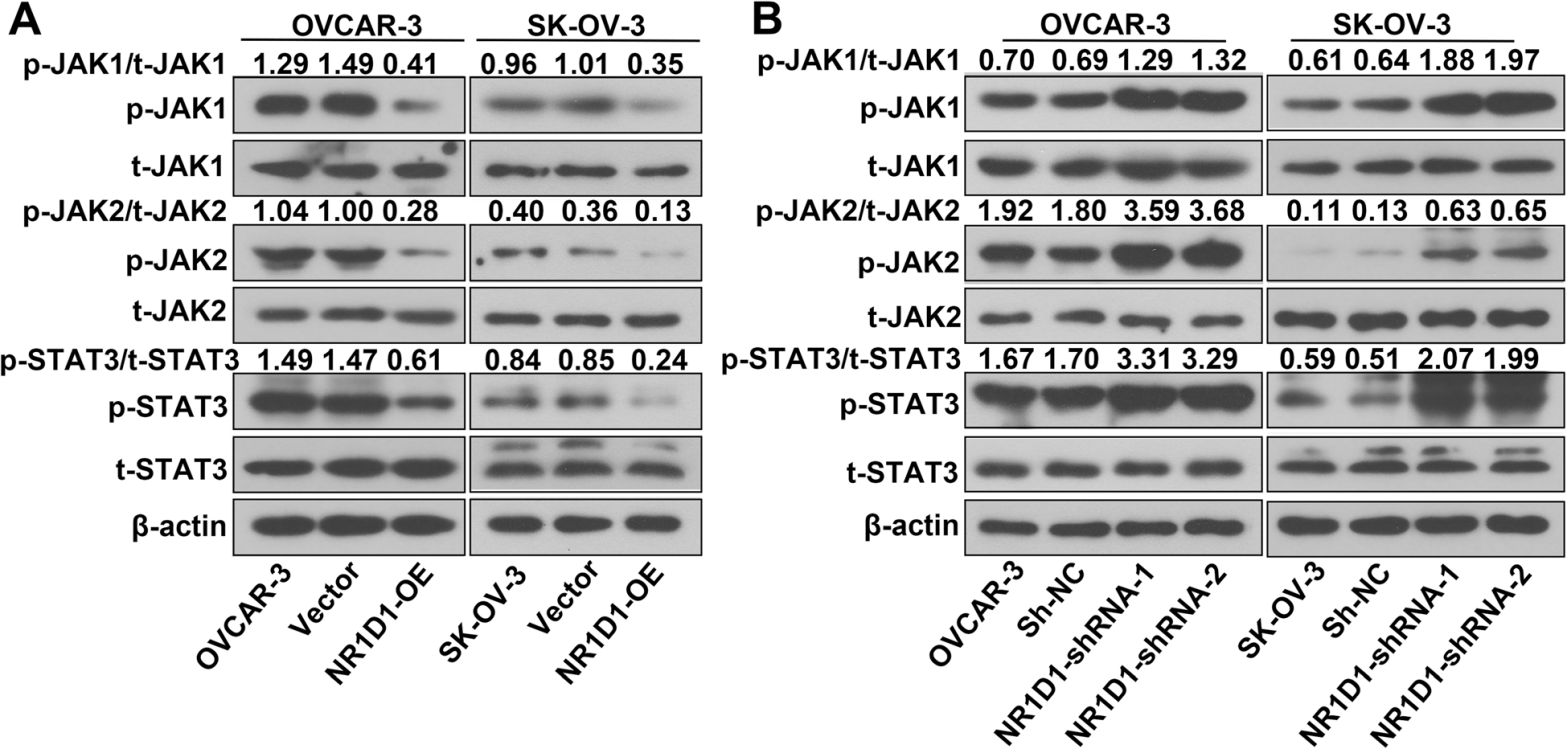
NR1D1 inhibited the activation of JAK/STAT3 signaling pathway. **A** After transfection with NR1D1 over-expression plasmid in OVCAR-3 and SK-OV-3 cells, the levels of phosphorylated JAK1, phosphorylated JAK2 and phosphorylated STAT3 were determined by western blot. **B** Western blot was performed to determine the levels of phosphorylated JAK1, phosphorylated JAK2 and phosphorylated STAT3 in NR1D1 silenced cells

The JAK/STAT3 signaling pathway plays a critical role in cancers. We found that in NR1D1 over-expressed cells, the levels of phosphorylated JAK1, JAK2 and STAT3 were decreased, while there were no significant changes in the levels of total JAK1, JAK2 and STAT3 (Fig. 3A). Consistently, in NR1D1 silenced cells, the levels of phosphorylated JAK1, JAK2 and STAT3 were increased, with no significant changes in the levels of total JAK1, JAK2 and STAT3 (Fig. 3B). These results revealed that NR1D1 inhibited the activation of JAK/STAT3 signaling pathway.

### NR1D1 suppressed the growth of ovarian cancer cells in vivo

**Fig. 4:**
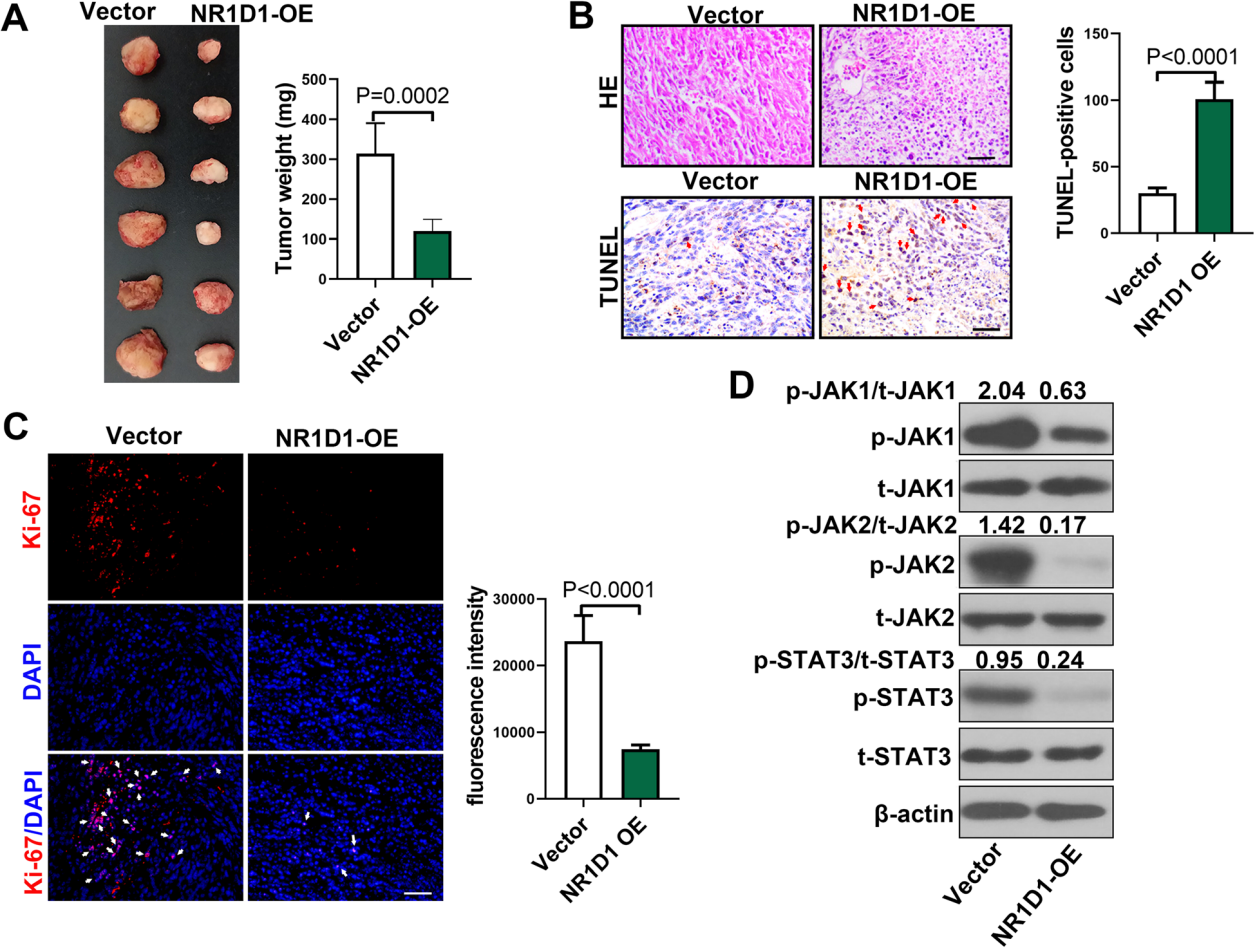
NR1D1 inhibited the growth of ovarian cancer cells in vivo. Ovarian cancer cells with stable transfection of NR1D1 over-expression plasmid were subcutaneously injected into the left flank of nude mice. Twenty-one days later, the tumors were obtained. **A** The images of tumors and tumor weight. **B** HE and TUNEL assay was performed to determine the histopathological changes and apoptosis in tumors. Typical images were presented. Scale bar = 50 μm. Red arrows indicated TUNEL positive cells. TUNEL-positive cells were counted. **C** The level of Ki-67 in tumors was determined by immunofluorescence. Scale bar = 50 μm. White arrows indicated Ki-67 positive cells. The fluorescence intensity of Ki-67 was examined. **D** The levels of phosphorylated JAK1, phosphorylated JAK2 and phosphorylated STAT3 in tumors with NR1D1 over-expression were determined with western blot

The role of NR1D1 in the growth of ovarian cancer cells was also explored in vivo. Tumors with NR1D1 over-expression were smaller than the controls (vector), with lighter tumor weights (Fig. 4A). Tumors with NR1D1 over-expression showed increased TUNEL staining with distinct regions of apoptosis/necrosis unlike tumors in the control group (Fig. 4B), which was consistent with the decreased tumor volumes. Besides, as determined by immunofluorescence, there was a decreased Ki-67 staining in NR1D1 over-expressed tumors compared with the controls (Fig. 4C). Furthermore, in tumors with NR1D1 over-expression, the levels of phosphorylated JAK1, JAK2 and STAT3 were decreased, whereas, the level of total JAK1, JAK2 and STAT3 showed no significant difference (Fig. 4D). These results suggested that NR1D1 suppressed the in vivo growth of ovarian cancer cells and activation of the JAK/STAT3 signaling pathway.

### NR1D1 inhibited the proliferation and induces apoptosis through up-regulating SOCS3

**Fig. 5:**
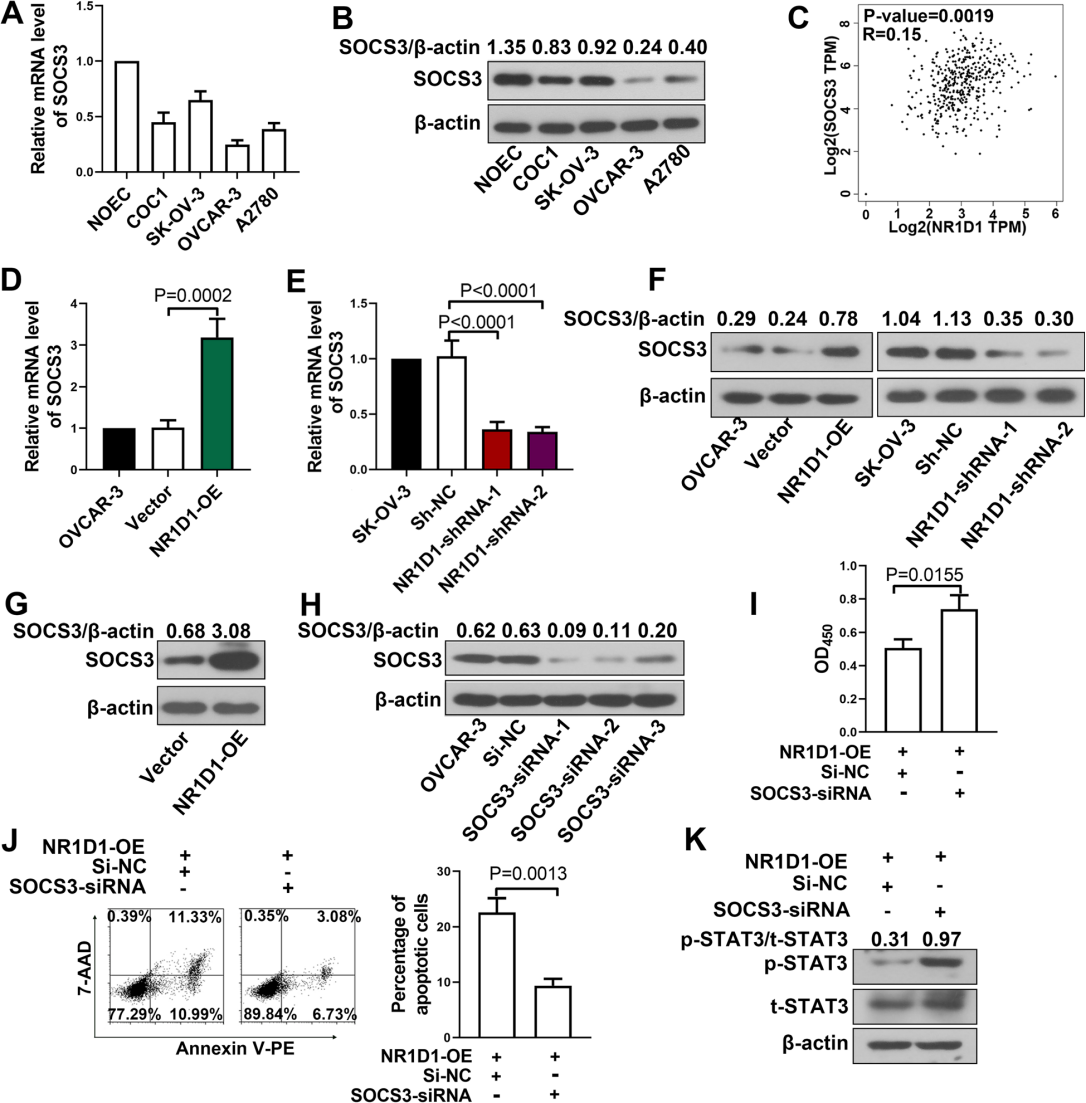
NR1D1 inhibited proliferation, induced apoptosis and suppressed the STAT3 signal through up-regulating SOCS3. **A-B** The level of NR1D1 in ovarian cancer cell lines COC1, SK-OV-3, OVCAR-3, A2780 and normal ovarian epithelial cell line (NOEC) was determined by quantitative real-time PCR and western blot. **C** Positive correlation between NR1D1 and SOCS3 in ovarian cancer according to the data from TCGA (*n* = 426). **D-E** The mRNA level of SOCS3 was determined by quantitative real-time PCR after transfection with NR1D1 over-expression plasmid or shRNAs. **F** The protein level of SOCS3 was determined by western blot after transfection with NR1D1 over-expression plasmid or shRNAs. **G** The level of SOCS3 in tumors with NR1D1 over-expression was determined by western blot. **H** Transfection efficiencies of SOCS3 siRNAs were determined by western blot. **I** After co-transfection with NR1D1 over-expression plasmid and SOCS3 siRNA, the cell viability was determined with CCK-8. **J** Apoptosis was determined by flow cytometry after co-transfection with NR1D1 over-expression plasmid and SOCS3 siRNA. **K** The level of phosphorylated STAT3 was determined by western blot in cells co-transfected with NR1D1 over-expression plasmid and SOCS3 siRNA. The results are presented as mean + SD

The level of SOCS3 in ovarian cancer cell lines was lower than that in NOEC, at both mRNA level and protein level (Fig. 5A-B). According to data from TCGA, NR1D1 was positively correlated with SOCS3, an inhibitor of the JAK/STAT3 signaling pathway (Fig. 5C). In NR1D1 over-expressed cells, the level of SOCS3 was increased, while in NR1D1 silenced cells, the SOCS3 level was decreased (Fig. 5D-F). On the other hand, in tumors with NR1D1 over-expressed, the level of SOCS3 was also increased (Fig. 5G). These results demonstrated that NR1D1 positively regulated the expression of SOCS3.

**Fig. 6:**
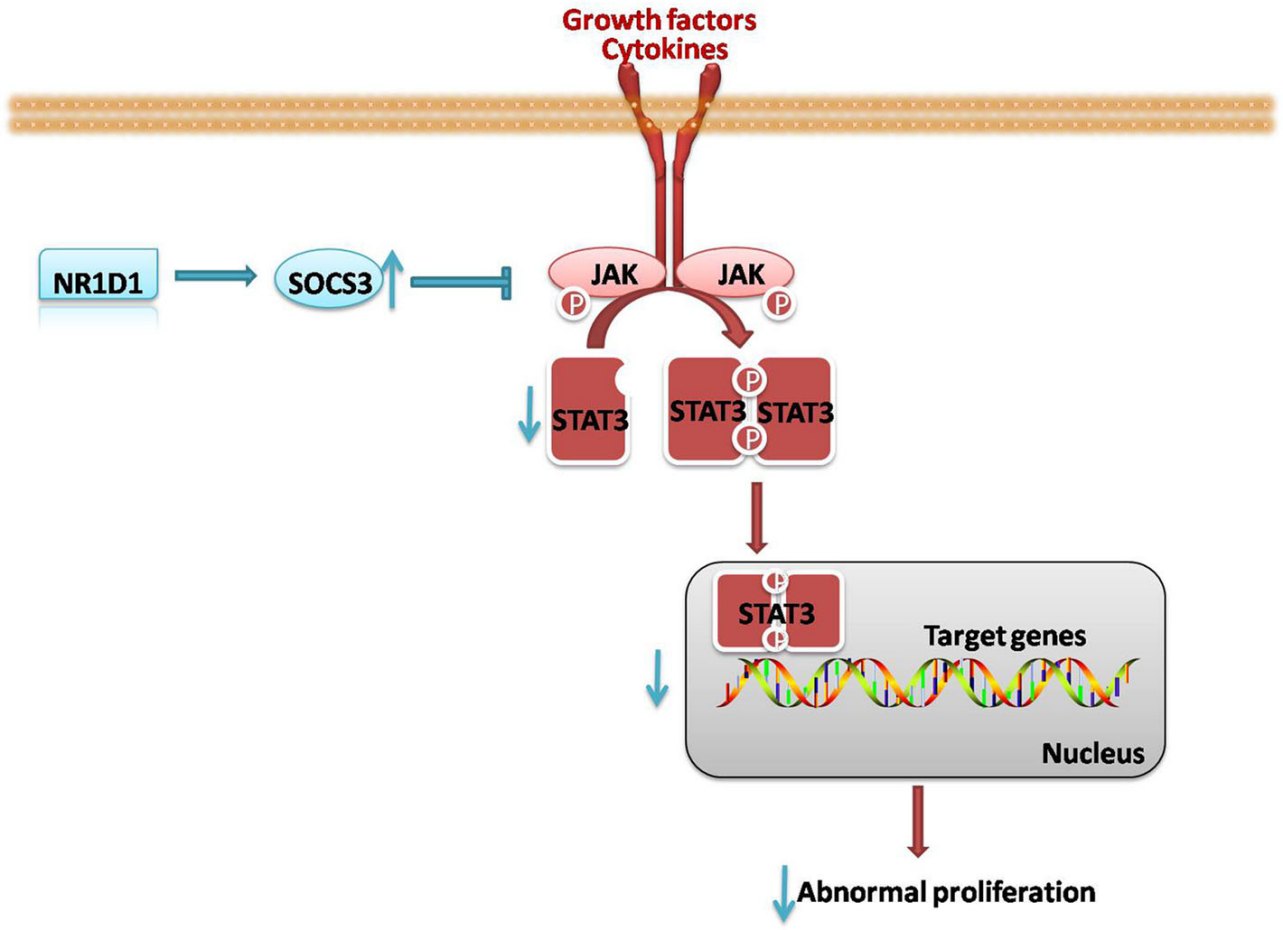
Schematic diagram: NR1D1 suppressed the growth of ovarian cancer through the JAK/STAT3 signaling pathway

Thereafter, SOCS3 siRNAs were introduced to explore whether NR1D1 performed its function through regulating SOCS3. First, the efficiencies of SOCS3 siRNAs were confirmed by western blot (Fig. 5H). Thereafter, SOCS3 siRNA was co-transfected into cells with NR1D1 over-expression, and then the cell viability, apoptosis and phosphorylation of STAT3 were detected. In cells co-transfected with NR1D1 over-expression plasmid and SOCS3 siRNA, the cell viability was higher (Fig. 5I) and the percentage of apoptotic cells was lower (Fig. 5J) than cells co-transfected with NR1D1 over-expression plasmid and si-NC. In addition, the level of phosphorylated STAT3 was increased in cells co-transfected with NR1D1 over-expression plasmid and SOCS3 siRNA (Fig. 5K). It was indicated that silencing SOCS3 abolished the function of NR1D1 over-expression on the proliferation and apoptosis of ovarian cancer cells as well as the activation of STAT3 signal (Fig. 6).

## Discussion

Data from TCGA showed that NR1D1 has a low expression level in ovarian cancer tissues, whereas, its role in ovarian cancer is not yet clear. In the present study, we explored the function of NR1D1 in ovarian cancer. Our study showed that NR1D1 suppressed the proliferation and induced apoptosis of ovarian cancer cells. Further study showed that NR1D1 inhibited the activation of JAK/STAT3 signaling pathway through positively regulating SOCS3. These data highlight a profound role of NR1D1 in the treatment of ovarian cancer. NR1D1 is also implicated in the regulation of circadian rhythm. Circadian rhythm disruption is reported to increase the cancer risk [25, 26]. Accordingly, the World Health Organization classified the shift-work, which was associated with disrupted circadian rhythm, as a possible carcinogen [27]. Moreover, circadian rhythm gene Bmal1, which was down-regulated in tongue squamous cell carcinoma (TSCC), suppressed the growth and metastasis of TSCC [28]. Circadian rhythm gene PER2, which had a low expression in ovarian cancer cells, repressed the growth of ovarian cancer cell SKOV-3 and enhanced their sensitivity to cisplatin [29]. Hence, abnormal expression of circadian rhythm genes has a close relationship with cancer risk and chemotherapy sensitivity [29–33].

Dysregulation of gene expression in cancers may contribute to the tumourgenesis. NR1D1 has a low level in ovarian cancer cell lines. Also, according to data from TCGA, NR1D1 has a low level in ovarian cancer tissues. Its low level is also associated with the poor survival of ovarian cancer patients at advanced stage. Hence, we wonder what the function of NR1D1 is in ovarian cancer cells. Over-expression of NR1D1 retarded the proliferation of ovarian cancer cells, while NR1D1 silencing accelerated their growth, suggesting that NR1D1 suppresses the growth of ovarian cancer cells. In addition, PCNA, which is closely related to cell proliferation and served as a biomarker of cell growth, was also reduced by NR1D1. These results provided additional evidence for our hypothesis that NR1D1 suppresses the growth of ovarian cancer. Besides, its in vivo growth was also repressed by over-expression of NR1D1, indicating that NR1D1 may act as a tumor suppressor. Consistently, down-regulation of NR1D1 promotes the proliferation of colon cancer cells [34], while activation of NR1D1 is lethal to cancer cells [6]. NR1D1 is also associated chemosensitivity [8]. Hence, we hypothesize that activation of NR1D1 may be beneficial to the treatment of ovarian cancer.

Cell cycle is a key factor that orchestrates the cell growth. Our study showed that NR1D1 arrested cell cycle at G1 phase and decreased the levels cell cycle-associated proteins, such as cyclins, indicating that cell cycle arrest induced by NR1D1 may contribute to its role in ovarian cancer growth. Consistently, activation of NR1D1 was also reported to reduce the level of cyclinA in breast cancer cells [7]. On the other hand, apoptosis was induced by NR1D1 over-expression, which may contribute to the tumor-suppressor role of NR1D1 in ovarian cancer. Apoptosis of cancer cells contributes to the therapeutic effects of anti-tumor drugs. NR1D1 is reported to be recruited to the damaged DNAs and inhibits their repair [8]. It also enhances the accumulation of ROS-induced DNA damage in breast cancer cells through the interaction with PARP1, thus increasing their sensitivity to oxidative stress [9]. As chemotherapeutic drugs for ovarian cancer treatment also promote the generation of ROS [35], we speculate that NR1D1 may also influence the chemoresistance of ovarian cancer cells. This speculation may be further confirmed in our future study.

Activation of the JAK/STAT3 signaling pathway contributes to the uncontrolled proliferation of cancer cells, including ovarian cancer [36]. Inhibition of the JAK/STAT3 signal suppresses the growth of ovarian cancer cells [37] and reduces their dissemination to the peritoneal cavity [19]. In our study, the JAK/STAT3 signaling pathway was inhibited by NR1D1 over-expression and enhanced by NR1D1 silencing, indicating that the JAK/STAT3 signal may be implicated in the role of NR1D1 in ovarian cancer cells. We wonder how NR1D1 influences the JAK/STAT3 signal in ovarian cancer cells. SOCS3 is an inhibitor of the JAK/STAT3 signaling pathway. The expression of SOCS3 is induced by excessive STATs activation, which in turn suppresses the bond of JAKs to receptors, thus suppressing their kinase activities as well as the phosphorylation of STAT3 [38]. On the other hand, SOCS3 promotes the ubiquitination and degradation of JAKs, thus reducing their stability [39]. As SOCS3 has a low expression level in ovarian cancer tissues [40] and positively correlated with the level of NR1D1, we speculate that NR1D1 may influence the JAK/STAT3 signaling pathway as well as ovarian cancer cell growth through modulating SOCS3. We found that NR1D1 positively regulated the expression of SOCS3. Additionally, silencing SOCS3 abolished the effect of NR1D1 over-expression on the proliferation and apoptosis of ovarian cancer cells, confirming that NR1D1 performs its role in ovarian cancer cells through modulating the expression of SOCS3. However, it is unclear how NR1D1 regulates the level of SOCS3. According to literature review, NR1D1 positively regulated the expression of transcription factor EB (TFEB) [41], while TFEB up-regulated the expression of SOCS3 [42]. Thus we speculated that NR1D1 may up-regulate the level of SOCS3 via TFEB. Other cytokines may also participate in the regulation of SOCS3 by NR1D1, and more researches are needed.

## Conclusions

Our study revealed that NR1D1 inhibited the activation of JAK/STAT3 signaling pathway through up-regulating SOCS3, thus suppressing proliferation and inducing apoptosis of ovarian cancer cells. These results indicate that NR1D1 may act as a tumor-suppressor in ovarian cancer cells, and provide basis for novel strategy of ovarian cancer treatment.

## Supplementary Information

**Additional file 1.** Original blot images.

## Data Availability

All data generated or analyzed during this study are included in this article.

## Abbreviations

NR1D1: Nuclear receptor subfamily 1 group D member 1
JAK: Janus kinase
STAT: Signal transducer and activator of transcription
SOCS3: Suppressor of cytokine signaling 3
TCGA: The Cancer Genome Atlas
FBS: Fetal bovine serum
PCNA: Proliferating cell nuclear antigen
HE: Hematoxylin-eosin
TFEB: Transcription factor EB
TUNEL: Terminal deoxynucleotidyl transferase-mediated dUTP nick end labeling
SD: Standard deviation
